# Endurance Training Increases the Running Performance of Untrained Men without Changing the Mitochondrial Volume Density in the Gastrocnemius Muscle

**DOI:** 10.3390/ijms231810843

**Published:** 2022-09-16

**Authors:** Jerzy A. Zoladz, Joanna Majerczak, Lukasz Galganski, Marcin Grandys, Justyna Zapart-Bukowska, Piotr Kuczek, Leszek Kołodziejski, Lucyna Walkowicz, Dorota Szymoniak-Chochół, Wincenty Kilarski, Wieslawa Jarmuszkiewicz

**Affiliations:** 1 Chair of Exercise Physiology and Muscle Bioenergetics, Faculty of Health Sciences, Jagiellonian University Medical College, Skawinska 8, 31-066 Krakow, Poland; 2 Laboratory of Mitochondrial Biochemistry, Department of Bioenergetics, Faculty of Biology, Adam Mickiewicz University, Uniwersytetu Poznańskiego 6, 61-614 Poznan, Poland; 3 Department of Physical Education, Faculty of Health Sciences, University of Applied Sciences in Tarnow, Mickiewicza 8, 33-110 Tarnow, Poland; 4 Department of Nursing, Faculty of Health Sciences, University of Applied Sciences in Tarnow, Mickiewicza 8, 33-110 Tarnow, Poland; 5 Department of Radiology and Imaging, John Paul II Hospital, Prądnicka 80, 31-202 Krakow, Poland; 6 Polish Academy of Science, Slawkowska 17, 31-018 Krakow, Poland

**Keywords:** critical power, heart rate, muscle fatigue, oxygen uptake, running

## Abstract

The activity and quantity of mitochondrial proteins and the mitochondrial volume density (Mito_VD_) are higher in trained muscles; however, the underlying mechanisms remain unclear. Our goal was to determine if 20 weeks’ endurance training simultaneously increases running performance, the amount and activity of mitochondrial proteins, and Mito_VD_ in the gastrocnemius muscle in humans. Eight healthy, untrained young men completed a 20-week moderate-intensity running training program. The training increased the mean speed of a 1500 m run by 14.0% (p = 0.008) and the running speed at 85% of maximal heart rate by 9.6% (p = 0.008). In the gastrocnemius muscle, training significantly increased mitochondrial dynamics markers, i.e., peroxisome proliferator-activated receptor gamma coactivator 1-alpha (PGC-1α) by 23%, mitochondrial transcription factor A (TFAM) by 29%, optic artrophy-1 (OPA1) by 31% and mitochondrial fission factor (MFF) by 44%, and voltage-dependent anion channel 1 (VDAC1) by 30%. Furthermore, training increased the amount and maximal activity of citrate synthase (CS) by 10% and 65%, respectively, and the amount and maximal activity of cytochrome *c* oxidase (COX) by 57% and 42%, respectively, but had no effect on the total Mito_VD_ in the gastrocnemius muscle. We concluded that not Mito_VD_ per se, but mitochondrial COX activity (reflecting oxidative phosphorylation activity), should be regarded as a biomarker of muscle adaptation to endurance training in beginner runners.

## 1. Introduction

Regular physical activity exerts a number of adaptive responses in various organs of the human body, and is considered an important factor in the prevention and treatment of many diseases [1]. Physical training over a relatively short period (weeks/months) increases human exercise capacity by enhancing the levels of various factors that limit physical performance [2,3]. In locomotor skeletal muscles, physical training can increase both mitochondrial protein content and mitochondrial enzyme activity, as shown in a classic study by Holoszy [4]. Subsequently, it has been demonstrated that endurance training markedly increases the amount of COX protein and its activity in the trained skeletal muscles in humans [5,6,7]. Interestingly, it has been also shown by Wibom et al. [7] that the magnitude of the training-induced increase in COX activity was closely related to the maximal mitochondrial ATP production rate studied ex vivo in humans. Adaptive muscle responses to exercise training were recently reviewed by Baldwin and Haddad [8]. Endurance training has been reported to increase mitochondrial volume density (Mito_VD_) in the vastus lateralis muscle of previously untrained individuals over a relatively short period of time [9,10,11,12,13]. Hoppeler et al. [14] showed that the central Mito_VD_ in the vastus lateralis muscle in humans was correlated with the maximal rate of oxygen consumption (VO_2max_, expressed in mL O_2_ × min^−1^ × kg body mass^−1^). This observation suggests that the training-induced increase in Mito_VD_ plays a key role in muscle adaptation to endurance training. However, the role of training-induced increases in muscle Mito_VD_ in the improvement of exercise capacity remains unclear [13,15,16].

An important question regarding the impact of physical training on human endurance performance involves which factors in the muscle (i.e., the increase in the maximal activity of citrate synthase (CS) and/or cytochrome *c* oxidase (COX), the increase in the amount of CS and/or COX proteins, or the increase in Mito_VD_) should be considered as the best measure of in vivo activity of mitochondrial oxidative phosphorylation (OXPHOS), and which of these factors results in training-induced enhancement of exercise performance.

Previous studies have shown that maximal in vitro muscle COX activity is the best measure of muscle OXPHOS activity [17,18]. Therefore, the training-induced increase in COX activity in the muscles should result in an increase in muscle metabolic stability and an increase in exercise endurance performance [19]. On the other hand, it was reported by Meinild Lundby et al. [13] that 6 weeks’ endurance training, which increased Mito_VD_ by ~55%, had no effect on functional changes in the OXPHOS capacity or COX activity in human muscle biopsy samples. Surprisingly, this considerable increase in Mito_VD_ was accompanied by a rather modest increase in VO_2max_ (~7%). These results therefore appear to question the role of training-induced COX activity and to weaken the importance of a training-induced increase in Mito_VD_ for increasing VO_2max_ and exercise tolerance in humans. 

The aim of our research was to expand the limited and ambiguous understanding of the role of training-induced increases in muscle Mito_VD_ in improving exercise performance in humans. We aimed to relate potential changes in Mito_VD_ after a 20-week endurance running program to running performance and changes in mitochondrial turnover markers, including markers of mitochondrial biogenesis and fission/fusion. Therefore, we focused on studying the training-induced changes in the following proteins: peroxisome proliferator-activated gamma 1-alpha receptor coactivator (PGC-1α), mitochondrial transcription factor A (TFAM), artrophy-1 (OPA1), mitochondrial cleavage factor (MFF), voltage-dependent anion channel 1 (VDAC1), as well as CS and COX protein content and maximal activity, and Mito_VD_ in gastrocnemius skeletal muscle in previously untrained young men. PGC-1α is a transcriptional coactivator that mediates many biological programs related to energy metabolism, including mitochondrial biogenesis [20,21]. TFAM, as a protein involved in the regulation of mtDNA transcription and replication [22,23,24], is considered a marker of skeletal muscle mitochondrial protein biogenesis [25,26]. VDAC1 is the most abundant protein in the mitochondrial outer membrane [27]. OPA1 enables the fusion of the inner mitochondrial membrane, having a significant impact on the mitochondrial cristae organization, whereas MFF is involved in the regulation of the mitochondrial fission process [28].

Based on previous studies, we hypothesized that training-induced increases in OXPHOS activity in the gastrocnemius muscle after weeks and/or months of training are sufficient to increase endurance running performance without the need to increase Mito_VD_ [17,18,29,30]. In addition, we discuss the possible mechanism by which the training-induced increase in gastrocnemius OXPHOS activity could improve running performance in humans. The present study is particularly warranted due to the huge popularity of endurance running as part of a healthy lifestyle.

## 2. Results

### 2.1. Physical Characteristics and HR_max_

The 20-week endurance running program reduced body mass by ~4%, from a mean of 84.05 ± 14.66 kg to 80.84 ± 10.13 kg, before and after training, respectively. Body mass index (BMI) decreased from 26.03 ± 3.68 kg × m^−2^ to 25.08 ± 2.61 kg × m^−2^, respectively, before and after training. The training-induced decrease in body mass and BMI was not statistically significant. Maximal heart rate (HR_max_) measured during the 1500 m run was 199.1 ± 7.6 beats per min.

### 2.2. Running Performance

**Figure 1:**
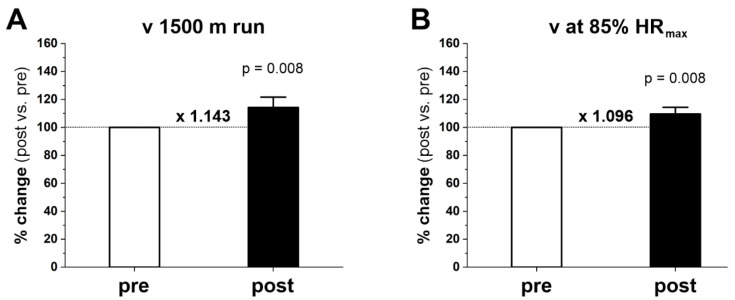
Effect of 20 weeks of endurance training on running performance over a distance of 1500 m. Mean running speed during the race (*v* 1500 m) (**A**) and the level of running speed reached at 85% maximal heart rate (*v* at 85% HR_max_) (**B**). Results are expressed as the percentage change after training (post) in relation to the status before training (pre), which is presented in the graph as 100%. Data are presented as the mean ± standard deviation (SD). The nonparametric Wilcoxon sign rank test with two-sided p-value is shown. Post- vs. prefold changes defined as the ratio of post-training value to pretraining value are marked as a multiple of the change, e.g., ×1.143 is a 1.143-fold increase.

Endurance running performance markedly increased after twenty weeks of training as determined by the levels of two variables: (i) mean running speed during the 1500 m race (m × s^−1^), and (ii) running speed at 85% HR_max_ (m × s^−1^). After the training period, the subjects completed the 1500 m time trial in a shorter time, on average, by 49.9 ± 28.7 s than before the training period. Thus, the mean speed of a 1500 m run (v 1500 m race) after the training period increased by 0.53 m × s^−1^ (i.e., by 14%) from 3.86 ± 0.43 m × s^−1^ before training to 4.39 ± 0.31 m × s^−1^ after training (p = 0.008) (Figure 1A). Running speed at 85% HR_max_ (v at 85% HR_max_) before training was 2.76 ± 0.37 m × s^−1^, and after training increased to 3.02 ± 0.42 m × s^−1^, or 9.6% (p = 0.008) (Figure 1B). Running speed at 85% HR_max_ corresponded to ~72% of the v 1500 m race before training and to ~69% after training. 

### 2.3. Mitochondrial Turnover

**Figure 2:**
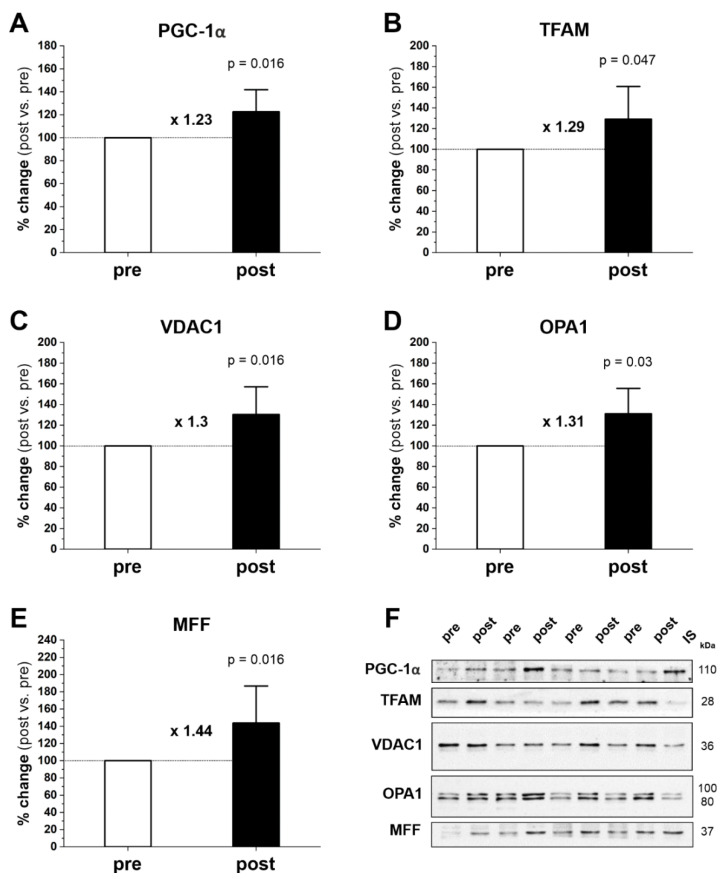
Effect of 20-week endurance training on the protein level of PGC1-α (**A**), TFAM (**B**), VDAC1 (**C**), OPA1 (**D**), and MFF (**E**) in human gastrocnemius muscle. Representative Western blots of 4 subjects and analysis of protein expression are shown (**F**). The internal standard (IS) is a human vastus lateralis muscle sample. The results are expressed as the percentage change after training (post) in relation to the status before training (pre), which is presented in the graph as 100%. Data are presented as the mean ± SD. The nonparametric Wilcoxon sign rank test with a two-sided p-value is shown. Post- vs. prefold changes defined as the ratio of post-training value to pretraining value are marked as a multiple of the change, e.g., ×1.23 is a 1.23-fold increase. Blot fragments are shown (framed). The entire blots and corresponding Ponceau detections are shown in Supplementary Figures S1–S5.

Mitochondrial dynamics are the continuous processes of mitochondrial biogenesis, fusion, and fission. We found that 20 weeks of endurance training significantly increased the protein level of PGC-1α by 23% (p = 0.016) (Figure 2A,F), TFAM by 29% (p = 0.047) (Figure 2B,F), VDAC1 by 30% (p = 0.016) (Figure 2C,F), OPA1 by 31% (p = 0.03) (Figure 2D,F), and MFF by 44% (p = 0.016) (Figure 2E,F).

**Figure 3:**
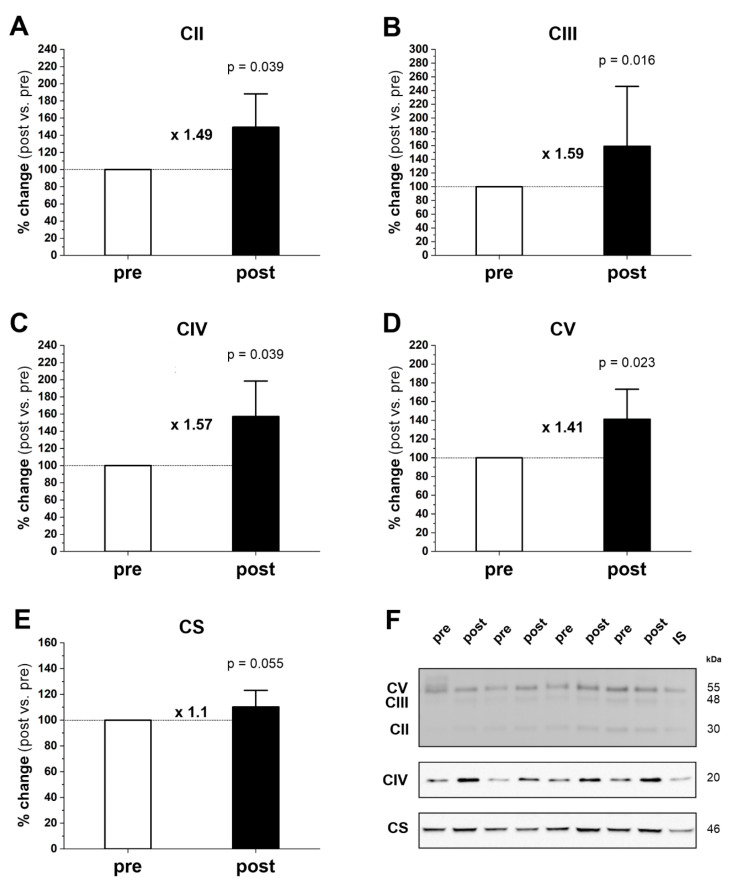
Effect of 20-week endurance training on the protein level of OXPHOS complexes, CII (subunit SDHB) (**A**), CIII (subunit UQCRC2) (**B**), CIV (subunit COXII) (**C**) and ATP synthase (CV, subunit ATP5A) (**D**), and CS (**E**) in human gastrocnemius muscle. Representative Western blots of four subjects are shown (**F**). The internal standard (IS) is a human vastus lateralis muscle sample. The results are expressed as percentage change after training (post) in relation to the status before training (pre), which is presented in the graph as 100%. Data are presented as the mean ± SD. The nonparametric Wilcoxon sign rank test with a two-sided p-value is shown. Post- vs. prefold changes defined as the ratio of post-training value to pretraining value are marked as a multiple of the change, e.g., × 1.49 is a 1.49-fold increase. Blot fragments are shown (framed). The entire blot and corresponding Ponceau detections are shown in Supplementary Figures S6–S8.

Furthermore, the 20-week endurance training affected the levels of mitochondrial OXPHOS proteins in the human gastrocnemius muscle. In particular, we found a significant increase in the following complexes: complex II (CII) (by 49%, p = 0.039), complex III (CIII) (by 59%, p = 0.016), and complex IV (CIV) (by 57%, p = 0.039), as well as complex V (CV) (by 41%, p = 0.023) (Figure 3A–F). Additionally, we noted a clear tendency (p = 0.055) for a higher CS content after the training program (by 10%) (Figure 3E,F).

### 2.4. Muscle COX and CS Activities

**Figure 4:**
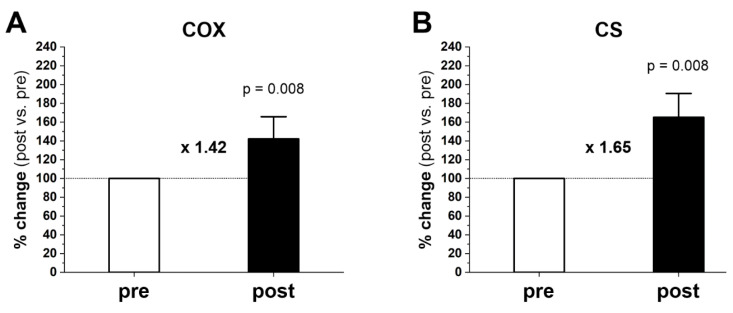
Training-induced changes in maximal cytochrome *c* oxidase (COX) activity (**A**) and maximal citrate synthase (CS) activity (**B**) in the human gastrocnemius muscle, studied at 37 °C. The results are expressed as a percentage of changes after training (post) in relation to the pretraining status (pre). Data are presented as the mean ± SD. The nonparametric Wilcoxon sign rank test with a two-sided p-value is shown. Post- vs. pre-fold changes defined as the ratio of post-training value to pretraining value are marked as a multiple of the change, e.g., × 1.42 is a 1.42-fold increase.

The maximal activity of COX in gastrocnemius muscle before training was 4842 ± 645 nmol O × min^−1^ × mg protein^−1^ and increased after training to 6829 ± 1261 nmol O × min^−1^ × mg protein^−1^ (p = 0.008). The maximal activity of CS before training was 8.40 ± 1.88 nmol 5.5’-di-thiobis-(2-nitrobenzoic) (TNB) × min^−1^ × mg protein^−1^, whereas after training it increased to 13.73 ± 3.10 nmol TNB × min^−1^ × mg protein^−1^ (p = 0.008). Figure 4 shows an ~42 and ~65% increase in COX (Figure 4A) and CS (Figure 4B) activity after training, respectively.

### 2.5. Mitochondrial Content in the Gastrocnemius Muscle

No significant effect of the 20-week endurance training was observed on the total Mito_VD_ in the gastrocnemius muscle or the volume density of its subsarcolemmal fraction (SS) (Table 1). However, a significant reduction in the volume density of the intermyofibrillar mitochondria (IMF) was observed (p = 0.017). 

## 3. Discussion

In the present study, we found that 20 weeks of moderate-intensity endurance training markedly improved running performance (Figure 1) and significantly increased protein levels of mitochondrial turnover, including mitochondrial biogenesis markers (PGC-1α and TFAM) and mitochondrial fusion (OPA1) and fission (MFF) markers in gastrocnemius muscle (Figure 2). The training-induced increase in PGC-1α and TFAM protein contents indicates that the endurance training was potent enough to enhance the biogenesis of skeletal muscle mitochondrial proteins [20,25,26]. Moreover, we found that endurance training significantly increased maximal COX and CS activities in gastrocnemius muscle (Figure 4A,B, respectively), along with increased COX protein content (COXII, p = 0.039) (Figure 3C), and a tendency to increase CS protein content (p = 0.055) (Figure 3E). These results indicate an increase in mitochondrial respiratory capacity in gastrocnemius muscle after training without significant changes in mitochondrial volume density (Mito_VD_) (Table 1). Thus, in human gastrocnemius muscle, a training-induced increase in muscle COX and CS protein content and activity precedes an increase in Mito_VD_.

It is well-established that endurance training improves the running performance of humans and attenuates muscle fatigue at a given running speed. Training-induced improvement in running performance can be achieved by: (i) increasing VO_2max_ and/or (ii) increasing running economy (i.e., a decrease in the oxygen cost of running at a given speed). Both factors alone can contribute to the enhancement of exercise performance after training. 

### 3.1. Training and VO_2max_

Using a simple calculation according to the formula proposed by Leger and Mercier [31], we estimated that the mean VO_2_ value achieved by the subjects during the 1500 m run before training would be ~48.7 mL O_2_ × min^−1^ × kg^−1^, whereas after training their performance would require the consumption of ~55.4 mL O_2_ × min^−1^ × kg^−1^ (i.e., ~14% more than that before training). The calculated pretraining and post-training VO_2_ values required for performing a 1500 m run can be considered a good approximation of the VO_2max_ of the runners [32]. According to this scenario, a post-training run of 1500 m would require an increase in VO_2max_ of the study participants by ~14%, which, taking into account previous studies that included endurance training of a similar duration (up to 6 months) [6,7,9,33], may be quite feasible.

Many factors may contribute to training-induced increases in VO_2max_ [2], among which, according to Joyner and Lundby [3], training-induced increases in stroke volume and total body hemoglobin appear to play a key role. Concerning this point, Korzeniewski and Rossiter [34] have recently postulated that training-induced increases in VO_2max_ are caused by the attenuation of P_i_ accumulation in muscles during exercise due to increased OXPHOS activity (and/or each-step activation intensity). Namely, based on cross-sectional comparisons of untrained and trained subjects, these authors argued that endurance training reduces the muscle P_i_ concentration in the final phase of fatiguing exercise (at VO_2max_), i.e., decreases the maximal muscle P_i_ concentration. On the other hand, Karlsson et al. [35] reported that training increased VO_2max_ in humans by 12% after 3 months and by 25% after 7 months of training; however, no significant differences were observed in muscle phosphocreatine concentration at VO_2max_ at both assessment time points. These experimental data challenge the outcome of the modeling reported by Korzeniewski and Rossiter [34], since the lowered maximal muscle P_i_ after the training, as postulated by these authors, should be accompanied by a lesser decrease in muscle PCr at the VO_2max_; however, as shown above [35], this was not the case. This illustrates that the maximal muscle P_i_ is not the ultimate factor that limits the VO_2max_ in humans [3]. 

### 3.2. Training and Running Economy

It is well-established that endurance-trained athletes have a higher running economy, meaning they consume less O_2_ at a specified running speed than untrained individuals [36]. This effect is especially visible at high running speeds [37] such that in untrained individuals there is a much greater magnitude of the slow component of the VO_2_ on-kinetics [19]. Physical training has been shown to attenuate the slow component and enhance the muscle power-generating capabilities at VO_2max_ [19]. For example, Majerczak et al. [38] reported that a 5-week endurance training program increased the power-generating capabilities at VO_2max_ by ∼7% (p = 0.002) in humans with no change in VO_2max_. These effects are due to a significant reduction in the nonlinearity in the VO_2_ vs. power output relationship in the heavy–severe exercise intensity domain. This effect was explained by an attenuation of the magnitude of the nonlinear increase in VO_2_ above the lactate threshold [39], thereby enabling the subjects to generate a higher power output at the same VO_2max_ [19]. The proposed explanation for the training-induced increase in muscle efficiency was a decrease in ATP usage at a given power output (i.e., a decrease in ATP/PO), due to the downregulation of SERCA2 pumps, which results in an enhancement of muscle metabolic stability at a given power output [38].

### 3.3. Mitochondrial Volume Density

Total Mito_VD_ in the gastrocnemius muscle tested before training was 9.78 ± 1.13% (Table 1) and was similar to the Mito_VD_ values found by others in the vastus lateralis muscle in humans [14,17]. As shown in Table 1, and in contrast to a previous study showing a significant increase of Mito_VD_ in the vastus lateralis [9,10,11,12,13], we found no significant effect of the endurance training program on Mito_VD_ in the gastrocnemius muscle. Our results indicate that the observed training-induced improvement in running performance is not related to Mito_VD_ in the gastrocnemius muscle. Furthermore, training-induced increases in muscle CS and COX activity were not accompanied by a change in Mito_VD_ in the gastrocnemius muscle, suggesting that such increases appear to be a poor marker of training-induced changes in the Mito_VD_ in gastrocnemius muscle. Interestingly, we observed a small but statistically significant decrease in the volume density of IMF mitochondria, but no change in the volume density of SS mitochondria (Table 1). This observed decrease in the volume density of IMF mitochondria in the gastrocnemius muscle after the training period is surprising, but may be related to the selective training-induced remodeling of this subpopulation of muscle mitochondria. The increase in mitochondrial turnover in the time-course of training has been reported as a part of a mechanism regulating the mitochondrial quality control in skeletal muscle [28]. In the present study, we found a significant increase in the markers of fusion (OPA1) and fission (MFF) of mitochondria (Figure 2D,E, respectively), indicating an enhancement in the turnover of mitochondrial proteins in trained gastrocnemius muscle. Moreover, we were surprised to find that the increased levels of markers of mitochondrial biogenesis and fusion/fission were accompanied by no increase in total Mito_VD_ in the trained muscle. This observation suggests that there was indeed an activation of the mitochondrial quality control process without Mito_VD_ enhancement at this stage of training.

### 3.4. Muscle COX and CS Activities

Based on current knowledge, the best “indirect” predictor of muscle OXPHOS activity is the maximal muscle COX activity [18]. This observation is in agreement with experimental data by Larsen et al. [17] showing a close correlation between muscle COX activity and muscle OXPHOS activity, defined as the maximal coupled respiration in permeabilized muscle fibers in humans. In the present study, we found a significant increase in maximal COX and CS activities (Figure 4), accompanied by an increase in COX protein and a strong tendency towards higher CS protein amount in the trained gastrocnemius muscle (Figure 3C,E). This 42% increase in COX activity in muscle could translate to a 42% increase in the muscle’s OXPHOS activity. The 42% increase in muscle maximal COX activity observed in this study is close to the 35% increase in maximal COX activity, as reported by Henriksson and Reitman [6] in the vastus lateralis muscle after 8–10 weeks of endurance training in humans, but much lower than the 78% increase in maximal COX activity reported by Wibom et al. [7] in the gastrocnemius muscle after just 6 weeks of a demanding endurance cycling training program in humans.

It is clear from our results that the training-induced improvement in endurance running performance did not require an increase in Mito_VD_ in the gastrocnemius muscle, which plays a key role in human locomotion such as walking or running [40]. Nevertheless, the training-induced improvement in running performance was accompanied by a pronounced increase in maximal muscle COX and CS activity (Figure 4). It seems possible that the observed increase in maximal COX and CS activity, in the absence of changes in Mito_VD_, may be accompanied by or due to a training-induced increase in mitochondrial cristae density, as previously proposed by Nielsen et al. [41], although this supposition has been questioned [42]. Alternatively, regardless of the increase in mitochondrial COX protein content, an increase in its activity through certain potent stimulating factors—such as increased catalytic efficiency due to increased affinity of the ferrocytochrome *c* subunit [43], and a training-induced decrease in NO in muscle tissue, leading to reduced COX inhibition [44]—may explain the increase in the gastrocnemius COX activity observed in the present study, in the absence of an increase in Mito_VD_.

### 3.5. The Effect of Muscle OXPHOS Activity on Running Performance

It has long been known that the oxygen cost of generating mechanical power (VO_2_/output power) at an intensity above the lactate threshold, or more strictly, above the critical power, is significantly higher than that below the lactate threshold or critical power [19,39,45], but the physiological reason for this effect is poorly understood. Based on published experimental data, our group has proposed that “…muscles could become less efficient when they approach the metabolic characteristics of fatigue, including a decrease in the Gibbs free energy of ATP hydrolysis, decreases in phosphocreatine and glycogen concentrations, and increases in [H^+^], [ADP_free_], [P_i_], [IMP], [NH_3_]” [46]. All these factors are involved in muscle fatigue [47,48,49]. Therefore, we have proposed that the increase in the VO_2_/power output ratio during high-intensity exercise is due to metabolic factors that induce muscle fatigue, and consequently reduces the efficiency of muscle contractions [19,46,50].

It is well-documented that exercise training attenuates the slow component of VO_2_ on-kinetics in humans and also increases power output at VO_2max_, even without changing VO_2max_ [19,38]. Karlsson et al. [35] reported that endurance training attenuates a decline in muscle phosphocreatine, as well as an increase in muscle lactate when cycling at a given power output. It should be mentioned, however, that for a long time the training-induced attenuation of muscle metabolic disturbances during exercise has not been considered to be the result of an increase in muscle OXPHOS activity.

Using experimental data and a computer model of OXPHOS [51], it was shown that an increase in OXPHOS activity decreases the level of muscle metabolites during exercise of a given power output [52]. Therefore, a training-induced increase in muscle efficiency during exercise above the lactate threshold or critical power, such as a 1500 m run, can largely be explained by the training-induced increase in OXPHOS activity. Therefore, we believe that increasing the activity of OXPHOS in the muscle leads to a decrease in muscle metabolites at a given power output, which results in a reduction in “additional ATP usage” and oxygen cost of work (i.e., a decrease in the VO_2_/power output ratio) [19,53]. These conditions help to reduce muscle fatigue at a given power output, increase critical power or critical speed, and achieve a higher power output at VO_2max_, even in the absence of an increase in VO_2max_ [19,38]. Therefore, the training-induced enhancement of OXPHOS enables the achievement of a higher power output/running speed before reaching critical muscle concentration levels of P_i_, ADP_free_, and H^+^–metabolites that are responsible for muscle fatigue [48,49,50]. This adaptive muscle response also appears to be important in preventing/slowing down aging-related lower limb muscle dysfunction, including sarcopenia-induced muscle weakness [54,55,56]. Nevertheless, this issue requires further research.

### 3.6. Other Potential Training-Induced Factors Important for Improving Running Speed

Other factors that may at least partially contribute to the observed improvement in running performance after training may be an increase in maximum cardiac output (Q) and blood oxygen-carrying capacity, resulting in an increase in VO_2max_ [12]. Furthermore, training-induced enhancement of mitochondrial efficiency (i.e., an increase in ATP/O ratio) [15,19] could also contribute to the lower oxygen cost of work [57] and in consequence allow for the generation of higher power output for a given VO_2_. Improvement in running economy, enhanced by a lower energy-consuming movement pattern when running, and weight loss (~4% in this study) can also be considered contributing factors to the observed training-induced improvement in running performance.

### 3.7. Summing Up

In the present study, we found that a 20-week endurance training program resulting in an increase in endurance running performance, accompanied by an increase in various markers of mitochondrial biogenesis (PGC1α, TFAM, VDAC, COX, and CS) and mitochondrial fusion/fission process (OPA1 and MFF) had no effect on total Mito_VD_ in trained gastrocnemius muscles. This indicates that the training-induced enhancement in the studied mitochondrial proteins content/activity precedes the expected increase in Mito_VD_. This does not exclude the possibility that a longer duration of applied training would result in an increase in total Mito_VD_ in the trained gastrocnemius muscle. It should be noted that we, like others [9,10,13,17], used the classical stereological approach to analyze two-dimensional data to estimate the three-dimensional parameters of muscle mitochondria (Mito_VD_), following the Weibel approach [58]. This allowed for a direct comparison of our results with previous reports on the effects of endurance training on skeletal muscle Mito_VD_. Nevertheless, it seems worth considering examining the impact of endurance training on skeletal muscle Mito_VD_ using a newly developed three-dimensional mitochondrial analysis [59,60], which could detect some earlier Mito_VD_ changes in trained muscles in the time-course of training.

## 4. Materials and Methods

### 4.1. Subjects

Eight healthy, non-smoking men with a mean age of 21.9 ± 0.8 years, height 179.4 ± 6.7 cm, body mass of 84.1 ± 14.7 kg, and BMI 26.03 ± 3.68 kg × m^−2^ were enrolled in the study. The participants were recruited through advertisement at the university campus with the following inclusion criteria: (1) age 20–25 years; (2) male gender; (3) no smokers; (4) good general health, i.e., no present illnesses and no taking medication; (5) no alcohol abuse; and (6) non-athletes. Before entering the study, candidates underwent standard medical evaluation and routine blood tests. Finally, they were interviewed by a doctor, medically examined (ECG and physical examination), and qualified/disqualified for this study. The candidates who positively passed this examination provided written informed consent for the study procedures. All subjects were untrained and reported rather low levels of voluntary physical activity (<2 h per week). In addition, they completed an eating habits survey (which was repeated after the training period) to exclude the potential impact of dietary changes on the study variables.

### 4.2. Running Performance

Endurance running performance was determined in a 1500 m run to exhaustion, conducted on a typical 400 m outdoor track. The subjects were instructed to complete the run as fast as possible at the speed of their choice. Based on the time needed to complete the 1500 m run to exhaustion, the mean running speed during the run was calculated (*v* 1500 m) and expressed in m × s^−1^. During the run, heart rate (HR) was continuously monitored and recorded throughout at 5 s intervals using a Polar RS400 heart rate monitor (Polar Electro Oy, Kempele, Finland). Moreover, an endurance submaximal running capacity for each runner was determined. Running capacity during the test was evaluated by determining running speed at 85% of HR_max_ (*v* at 85% HR_max_). This assessment was made on the basis of the linear relationship between running speed and HR, which was established for each subject during a running field test, as described previously by Zoladz et al. [61].

### 4.3. Muscle Biopsy and Analyses

Muscle biopsies were taken before and after the endurance training program. The second muscle biopsy (i.e., post-training) was obtained 74.0 ± 2.8 h after the last training session. Muscle biopsies were taken under local anesthesia (1% Lignocainum Hydrochloricum, Polfa, Warszawa, Poland) from the right gastrocnemius muscle with the use of a 2 mm biopsy needle (Pro-Mag^™^ I 2.2, Angiotech, Vancouver, BC, Canada). In the present study, we took a gastrocnemius muscle biopsy, as this muscle plays a key role in human locomotion such as walking and running [40]. The samples were immediately frozen in liquid nitrogen and used for further assessments of enzyme activity. The sample preparation was performed at 4 °C. After weighing, muscle biopsies (8–20 mg) were placed in a buffer consisting of 0.32 M sucrose, 1 mM (ethylenediaminetetraacetic acid, disodium salt) EDTA, and 10 mM Tris HCl, pH 7.4 (SET) and homogenized with a Polytron homogenizer (3 × 2 s). After a short (30 s) spin down of unbroken cells and cell debris, the supernatant was collected for the determination of CS and COX activity. The total protein concentration of the samples was determined by the Bradford method [62].

### 4.4. Muscle Mitochondrial Protein Content

Protein extraction from human gastrocnemius muscle was performed using the Complete Mammalian Proteome Extraction Kit (Calbiochem, Cat#539779). Protein concentrations in the muscle-derived extracts were measured using a Qubit fluorometer (Thermo Fisher Scientific™, Waltham, MA, USA), and the proteins were subsequently separated on a polyacrylamide gel. Equal amounts of total protein were loaded on the gels. The protein extract from vastus lateralis muscle of a young, healthy, non-smoking, untrained male (age: 25; height: 192.5 cm; weight: 73.5 kg; BMI: 19.83 kg × m^−2^; VO_2max_: 38.3 mL × min^−1^ × kg^−1^) was used for the internal standard to eliminate any differences between the gels due to unequal transfer. After transfer to a nitrocellulose membrane, detection of the protein bands on Western blot was performed using Ponceau S staining to ensure the equal loading and transfer of proteins. The following primary antibodies from ABCAM Cambridge, UK were used: anti-CS (46 kDa; Cat#ab96600), anti-TFAM (28 kDa; Cat#ab131607), anti-VDAC-1 (36 kDa; Cat#ab14734), anti-PGC-1α (110 kDa; Cat#ab54481), anti-MFF (37 kDa; Cat#ab81127), anti-COX (subunit COXII, 20 kDa; ab110413), and anti-total OXPHOS Cocktail (Cat#ab110413), which contained antibodies against subunits of complex II (CII, subunit SDHB, 30 kDa), CIII (subunit UQCRC2, 48 kDa) and CV (ATP5A, 55 kDa). We used also anti-OPA1 (100 and 80 kDa; Cat#612607) primary antibodies from BD Biosciences, Franklin Lakes, NJ, USA. Data were imaged using GeneGnome Syngene XRQ-NPC, and GeneTools Syngene analysis software was used for densitometric analysis (Syngene Bio Imaging, Cambridge, UK). The optical density values obtained for proteins detected in human skeletal muscle samples were normalized to an internal standard, and Ponceau staining was subsequently performed as previously described [63,64]. All densitometric data are presented as the percentage change in the level of protein after training (post), in relation to the pretraining status (pre), which is expressed as 100%.

### 4.5. Maximal CS and COX Activities

The maximal activities of CS and COX were determined as previously described [18]. The maximal activity of CS was measured spectrophotometrically in a 1 mL reaction mixture containing 100 μM Tris, pH 8.0, 100 μM acetyl-CoA, 100 mM 5.5′-di-thiobis-(2-nitrobenzoic) (TNB), 0.1% Triton X-00, and 25–60 μg of homogenate supernatant protein. The reaction was started with 100 µM oxaloacetate and monitored at 412 nm for 3 min at 37 °C. The activity of CS is expressed as nmol TNB × min^−1^ × mg protein^−1^.

The maximal COX activity was measured polarographically using a Clark-type oxygen electrode (Hansatech Instruments Ltd., King’s Lynn, UK) in 0.7 mL of incubation medium (37 °C) containing 0.17 M sucrose, 10 mM Tris HCl (pH 7.2), 2.5 mM KH_2_PO_4_, 4 mM MgCl_2_, and 0.2% bovine serum albumin (BSA), with 10–25 µg of supernatant protein. The reaction mixture contained antimycin A (3 µg × mL^−1^), 8 mM ascorbate, 0.08% cytochrome *c*, and up to 2.5 mM *N*,*N*,*N′N′*-tetramethyl-*p*-phenylenediamine (TMPD). Finally, 1 mM cyanide was added. The cyanide-sensitive rates reflect the level of COX activity (expressed in nmol O × min^−1^ × mg of protein^−1^).

### 4.6. Muscle Morphometry

**Figure 5:**
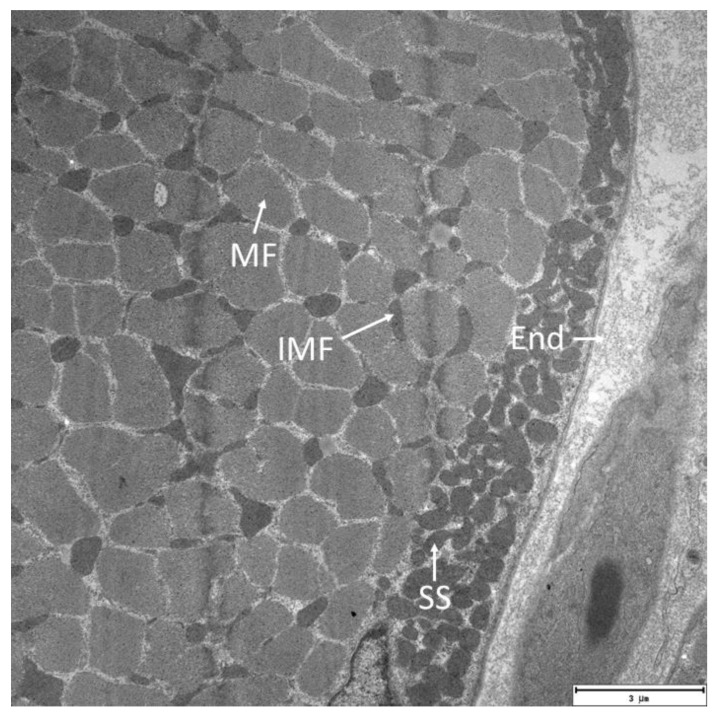
Representative electron micrograph of human gastrocnemius muscle from a subject participating in the present study. The electron microscopy image depicts subsarcolemmal (SS) and intermyofibrillar (IMF) mitochondria. MF, muscle fiber; End, endomysium.

Muscle biopsy samples were prepared for electron microscopy as previously described [30,53]. The relative number of mitochondria was determined by the stereological method [58], slightly modified for the purposes of the experiment. The relative volume of mitochondria is expressed as a percentage per cross-sectional area of a muscle fiber photographed under electron microscopy at 3000 × magnification. Three biopsies were randomly taken from each individual and analyzed after appropriate processing. For the stereological analysis, a minimum of 15 fibers were photographed at a magnification of 3000 ×. Two zones of muscle fiber cross-sections were analyzed: the inner zone, in which the intermyofibrillar mitochondria were counted, and the peripheral (subsarcolemmal) region, primarily adjacent to the capillary vessel. Figure 5 shows an electron micrograph of gastrocnemius fibers illustrating the subsarcolemmal (SS) and intermyofibrillar (IMF) mitochondria.

### 4.7. Endurance Training Program 

After completing all exercise running tests, subjects participated in the 20-week endurance training program, supervised by one of the authors. The training program consisted of an average of 3 running sessions per week performed at an intensity corresponding to 80–85% of HR_max_, which was determined during the pretraining 1500 m run-time trial. For the first 5 weeks of training, the running sessions were 30 min in duration, after which (from the beginning of the 6th week to the end of the 20th week) the duration of the run was extended to 40 min. Moreover, prior to each running session, participants completed the standardized warm-up protocol, comprised of 15 min of jogging, multidirectional movements, coordination exercises and dynamic stretching, followed by 5 acceleration runs (80 m long) performed at submaximal speed (~5 m × s^−1^). The training program began at the beginning of December and ended in the middle of March. During this period, the subjects completed on average a total of 60 training sessions and 37.5 h of running. Each training session was monitored using a heart rate monitor (Polar RS400, Polar Electro Oy, Kempele, Finland). The training sessions were conducted in the surroundings of a track and field stadium to avoid running on hard surfaces. During the 20 weeks of endurance running training (partly during the winter period) some subjects experienced mild cold. Two subjects had short-term musculoskeletal problems, one of whom suffered from knee pain, and the other from pain in the shin. After withdrawing from training (~1 week pause) and following a recommendation to avoid hard surfaces and run only on soft surfaces, these problems disappeared. During the training period, subjects were instructed to maintain their normal diet and to refrain from taking any nutritional supplements or consuming alcohol.

### 4.8. Statistics 

The results from this study are expressed as the mean ± standard deviation (SD). The statistical significance of the differences for paired samples was tested using the nonparametric Wilcoxon test, and nonasymptotic, exact, two-sided p-values are presented. The nonparametric test with the exact p-value was used because of the relatively small sample size (*n* = 8). p-values < 0.05 were considered statistically significant. Statistical analyses of the data were carried out using Origin2022 v 9.9 software (OriginLab Corporation, Northampton, MA, USA).

## 5. Conclusions

In the present study, we found that the training-induced improvement in endurance running capacity in previously untrained subjects did not require an increase in Mito_VD_ in the gastrocnemius muscle. In light of previous research, we postulate that the increase in COX activity (by 42%) observed in this study may have led to the considerable improvement in running performance. Such improvement can be achieved by decreasing exercise-induced changes in muscle metabolite concentrations at a given running speed, including a smaller decrease in muscle phosphocreatine and a smaller increase in muscle ADP_free_, H^+^, and P_i_. This adaptive muscle response can improve running economy, attenuate muscle fatigue at a given running speed, and increase running speed at VO_2max_. Finally, we postulate that measurements of maximal COX activity in muscles could be a useful biomarker of the adaptation of muscle mitochondria during endurance training in humans.

## Figures and Tables

**Table 1:** Mitochondrial content (% volume) in gastrocnemius muscle of the studied subjects before and after endurance training.

Status	Volume Density of Total Mitochondria in %	Volume Density of IMF Mitochondria in %	Volume Density of SS Mitochondria in %
Before training	9.78 ± 1.13	6.32 ± 1.04	3.30 ± 0.47
After training	8.54 ± 0.81 n.s.	5.14 ± 0.64 (p = 0.017)	2.94 ± 0.79 n.s.

Data are presented as the mean ± SD. SS, subsarcolemmal mitochondria; IMF, intermyofibrillar mitochondria; n.s., not significant.

## Data Availability

The datasets used and/or analyzed during the study are available from the corresponding author on reasonable request.

## Supplementary Materials

The following supporting information can be downloaded at: https://www.mdpi.com/article/10.3390/ijms231810843/s1.
