# Supplementary material for: Endurance Training Increases the Running Performance of Untrained Men without Changing the Mitochondrial Volume Density in the Gastrocnemius Muscle
Source: Int J Mol Sci. 2022 Sep 16;23(18):10843. doi: 10.3390/ijms231810843 (PMC9503714; doi:10.3390/ijms231810843)
Supplement: Supplementary file 1 [file ijms-23-10843-s001.zip › ijms-1888896-supplementary.pdf]

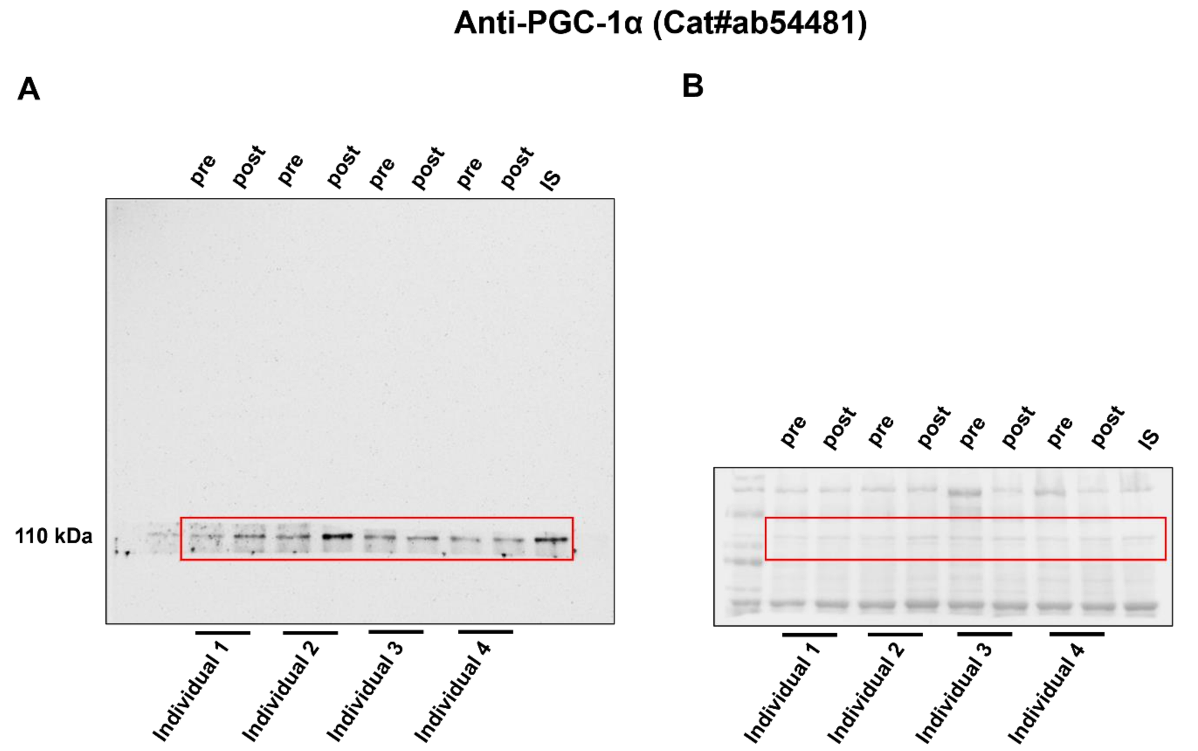

Figure S1. Representative immunoblot demonstrating detection of PGC-1α (Cat# ab54481, Abcam, Cambridge, UK) in human gastrocnemius muscle of 4 individuals (**A**). The red frame indicates the fragment of membrane which is presented in the main manuscript in Fig. 2F. Ponceau S staining of the membrane demonstrating total protein level in the samples (**B**). The internal standard (IS) is a human vastus lateralis muscle sample. The first lane contains mixture of all samples and molecular marker.

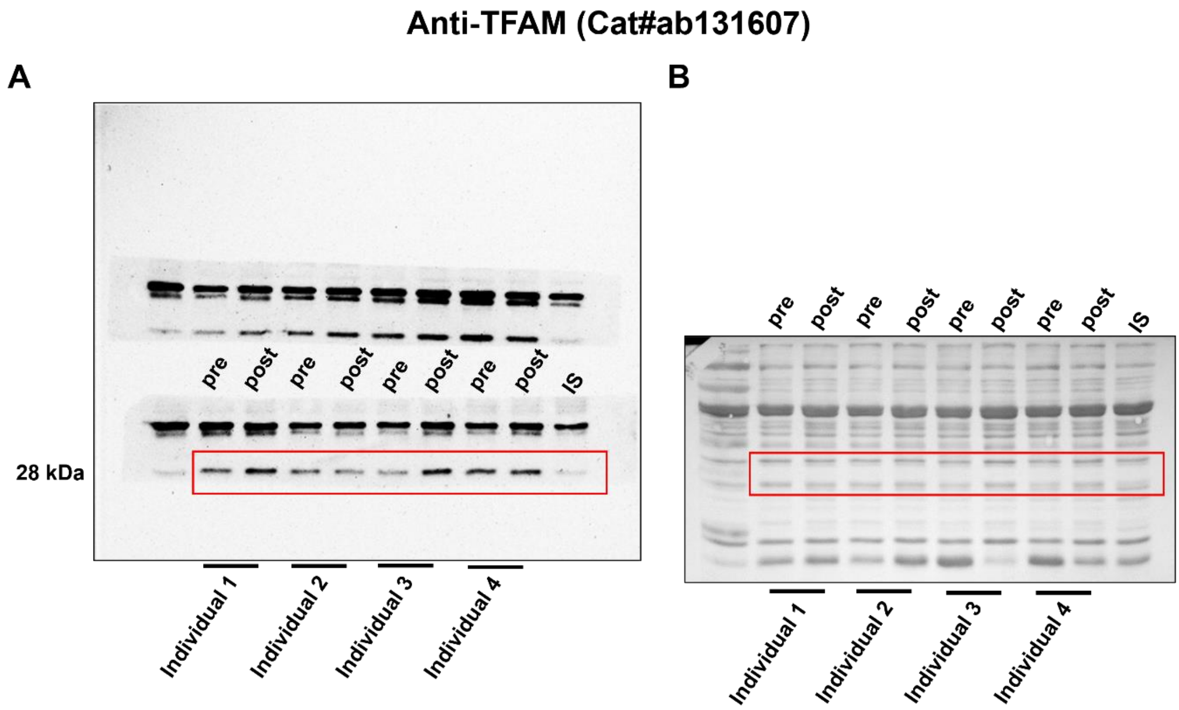

Figure S2. Representative immunoblot demonstrating detection of TFAM (Cat#ab131607, Abcam, Cambridge, UK) in human gastrocnemius muscle of 4 individuals (**A**). The red frame indicates the fragment of membrane, which is presented in the main manuscript in Fig. 2F. Ponceau S staining of the membrane demonstrating total protein level in the samples (**B**). The internal standard (IS) is a human vastus lateralis muscle sample. The first lane contains mixture of all samples and molecular marker.

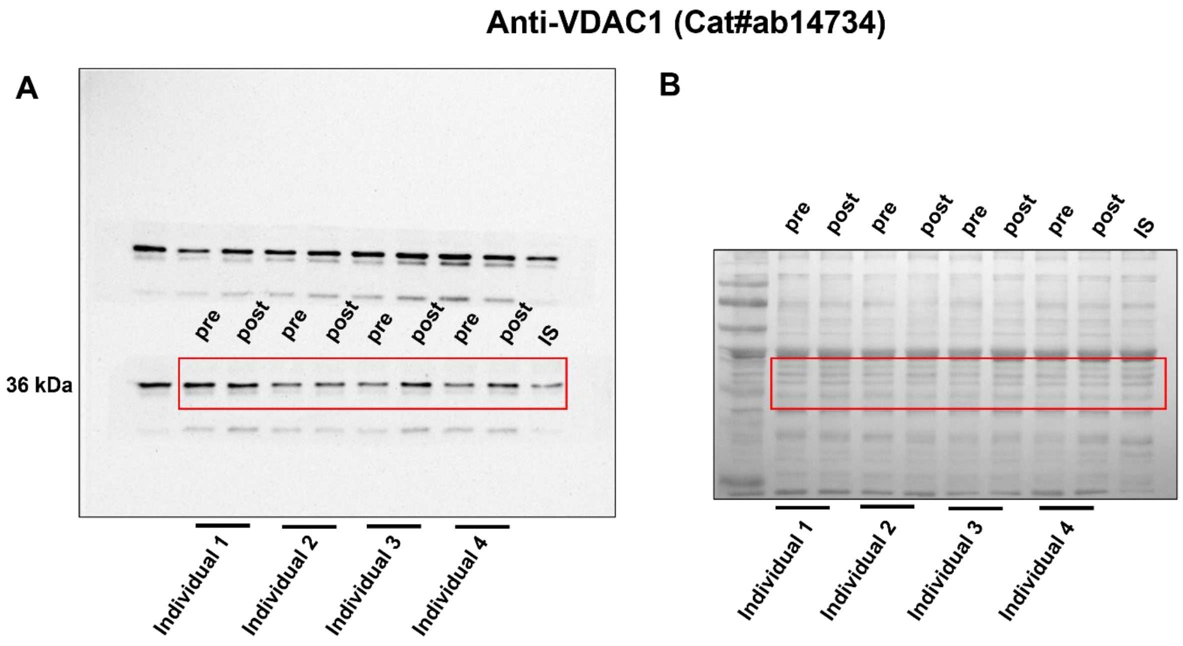

Figure S3. Representative immunoblot demonstrating detection of VDAC1 (Cat#ab14734, Abcam, Cambridge, UK) in human gastrocnemius muscle of 4 individuals (**A**). The red frame indicates the fragment of membrane which is presented in the main manuscript in Fig. 2F. Ponceau S staining of the membrane demonstrating total protein level in the samples (**B**). The internal standard (IS) is a human vastus lateralis muscle sample. The first lane contains mixture of all samples and molecular marker.

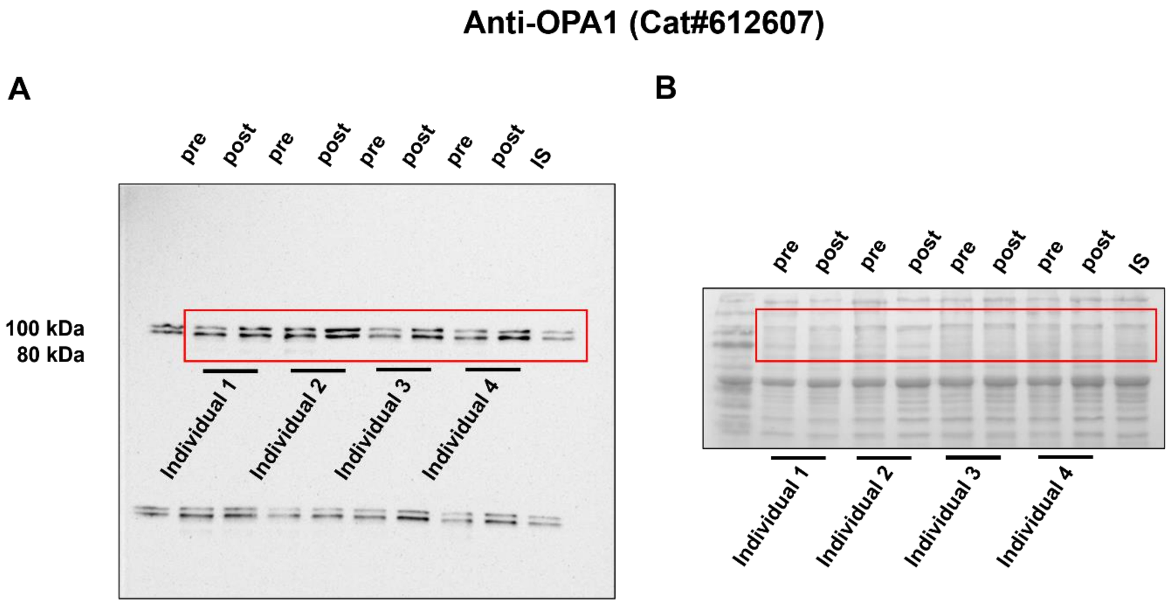

Figure S4. Representative immunoblot demonstrating detection of OPA1 (Cat#612607, BD Biosciences, Franklin Lakes, NJ, USA) in human gastrocnemius muscle of 4 individuals (**A**). The red frame indicates the fragment of membrane which is presented in the main manuscript in Fig. 2F. Ponceau S staining of the membrane demonstrating total protein level in the samples (**B**). The internal standard (IS) is a human vastus lateralis muscle sample. The first lane contains mixture of all samples and molecular marker.

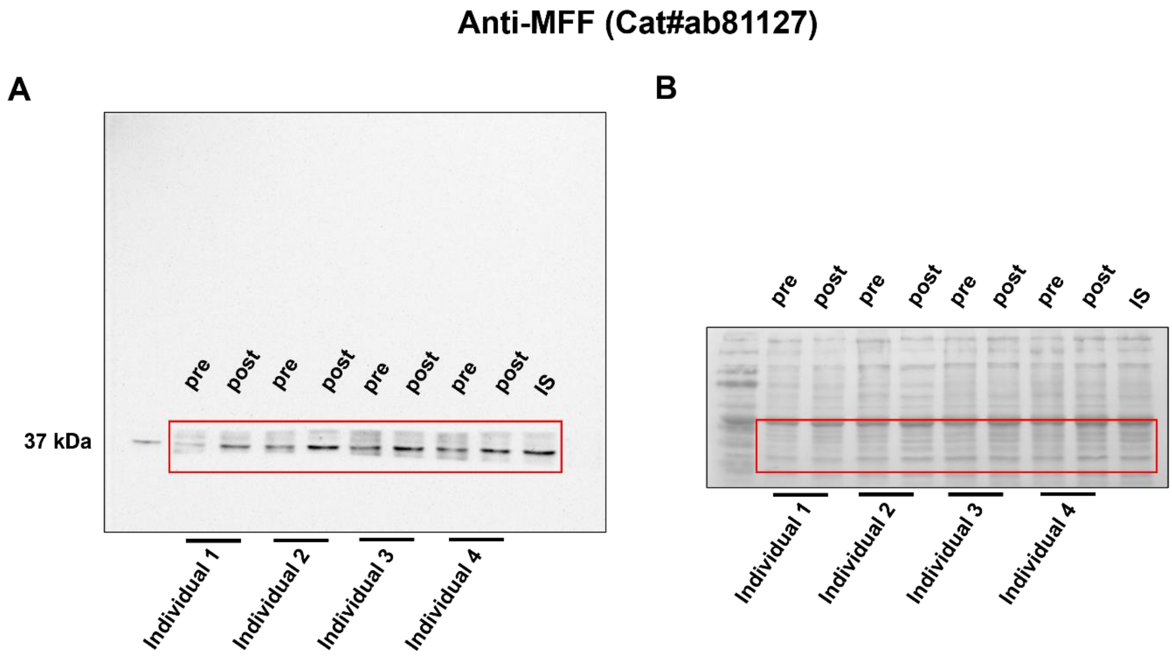

Figure S5. Representative immunoblot demonstrating detection of MFF (Cat#ab81127, Abcam, Cambridge, UK) in human gastrocnemius muscle of 4 individuals (**A**). The red frame indicates the fragment of membrane which is presented in the main manuscript in Fig. 2F. Ponceau S staining of the membrane demonstrating total protein level in the samples (**B**). The internal standard (IS) is a human vastus lateralis muscle sample. The first lane contains mixture of all samples and molecular marker.

Anti-total OXPHOS Cocktail (Cat#ab110413)

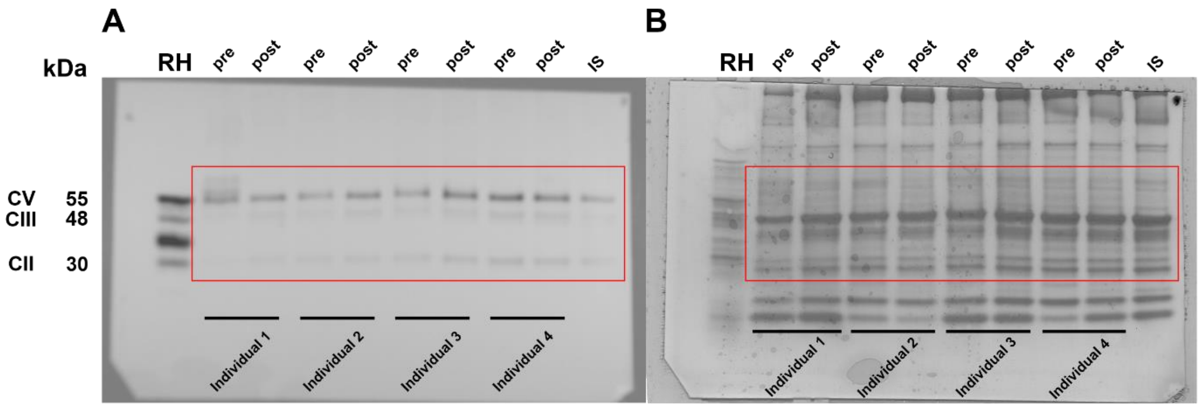

Figure S6. Representative immunoblot demonstrating detection of OXPHOS complexes (Cat#ab110413, Abcam, Cambridge, UK): CII (subunit SDHB), CIII (subunit UQCRC2) and ATP synthase (CV, subunit ATP5A) in human gastrocnemius muscle of 4 individuals (**A**). The red frame indicates the fragment of membrane, which is presented in the main manuscript in Fig. 3F. Ponceau S staining of the membrane demonstrating total protein level in the samples (**B**). The first lane contains a rat heart mitochondrial lysate (RH) as the positive control (ab110341). The internal standard (IS) is a human vastus lateralis muscle sample.

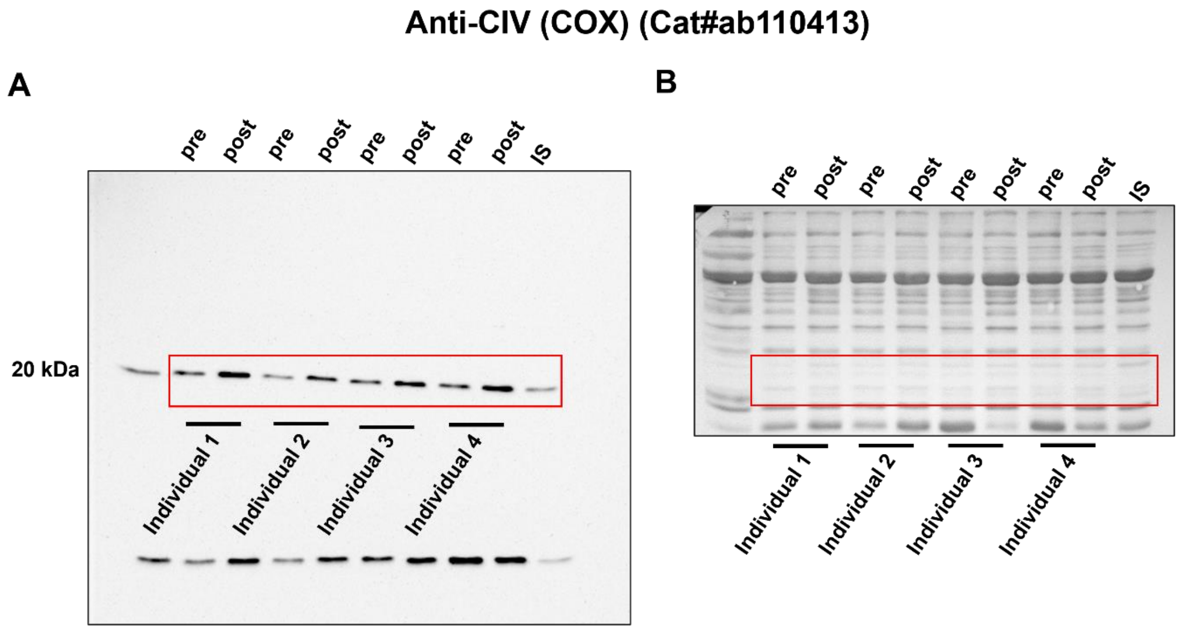

Figure S7. Representative immunoblot demonstrating detection of CIV (COX) (Cat#ab110413, Abcam, Cambridge, UK) in human gastrocnemius muscle of 4 individuals (**A**). The red frame indicates the fragment of membrane, which is presented in the main manuscript in Fig. 3F. Ponceau S staining of the membrane demonstrating total protein level in the samples (**B**). The internal standard (IS) is a human vastus lateralis muscle sample. The first lane contains mixture of all samples and molecular marker.

Anti-CS (Cat#ab96600)

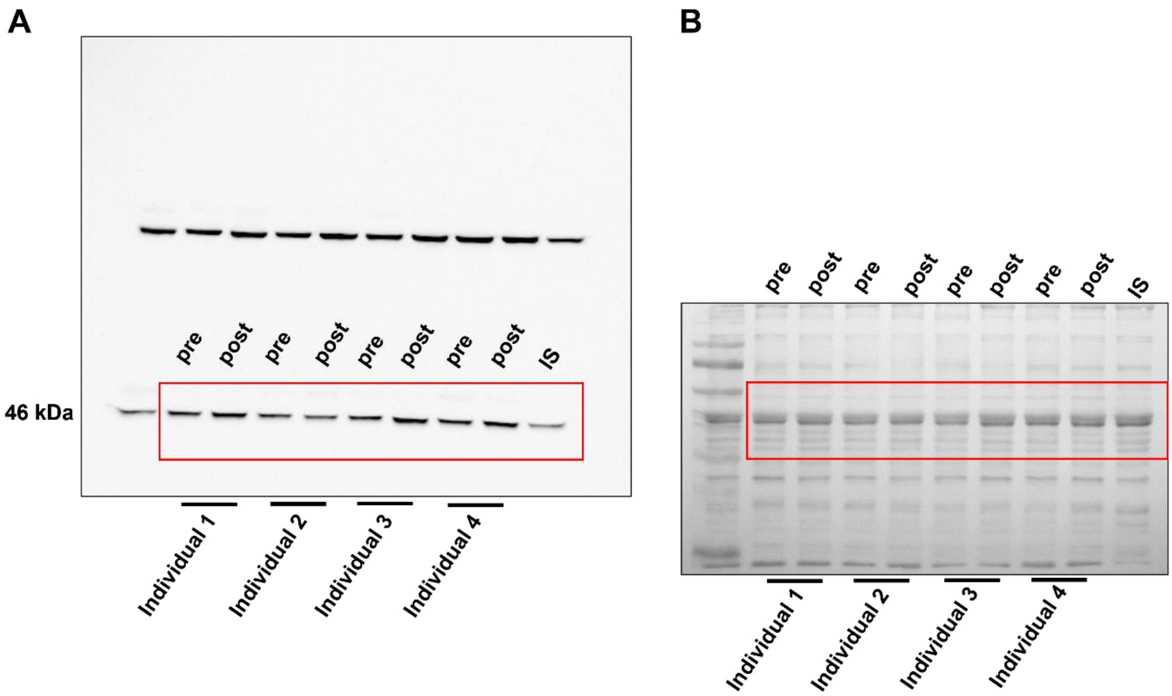

Figure S8. Representative immunoblot demonstrating detection of CS (Cat#ab96600, Abcam, Cambridge, UK) in human gastrocnemius muscle of 4 individuals (**A**). The red frame indicates the fragment of membrane which is presented in the main manuscript in Fig. 3F. Ponceau S staining of the membrane demonstrating total protein level in the samples (**B**). The internal standard (IS) is a human vastus lateralis muscle sample. The first lane contains mixture of all samples and molecular marker.
